# Real time endobronchial ultrasound transbronchial needle aspiration for the diagnosis of tuberculous intrathoracic lymphadenopathy: Saudi Arabian Western region experience

**DOI:** 10.15537/smj.2023.44.2.20220434

**Published:** 2023-02

**Authors:** Ahmed A. Aljohaney

**Affiliations:** *From the Department of Internal Medicine, Faculty of Medicine, King Abdulaziz University, Jeddah, Kingdom of Saudi Arabia.*

**Keywords:** endoscopes, ultrasonography, lymph nodes, mediastinum, thorax, safety, efficiency

## Abstract

**Objectives:**

To assess the utility of endobronchial ultrasound-guided transbronchial needle aspiration (EBUS-TBNA) for presumptive tuberculosis (TB) patients with intrathoracic enlarged lymph nodes in a country with low to moderate TB incidence.

**Methods:**

Thirty-one patients with clinical features of TB and intrathoracic lymphadenopathy, who had EBUS-TBNA sampling and final confirmation of intrathoracic TB lymphadenopathy, were retrospectively reviewed over an 8-year period. Routine clinical and laboratory evaluations including computerized tomography scans were performed before the EBUS-TBNA. Sociodemographic characteristics, clinical profile, pathological, and microbiological findings were collected.

**Results:**

The EBUS-TBNA confirmed TB diagnosis in 26 (83.9%) subjects with a consistent pathological finding or positive culture of *Mycobacterium tuberculosis*. Pathological analysis had findings consistent with TB in 25 (80.6%) patients. Culture of the EBUS-TBNA sample was positive for *Mycobacterium tuberculosis* in 12 (38.7%) patients. Other supportive investigations like purified protein derivative (PPD) skin test was positive in 28 (90.3%) participants. Overall, the sensitivity of the EBUS-TBNA alone was 83.9%. No complications were recorded during the procedure. The EBUS-TBNA aspirate culture positivity was significantly related to having a larger size lymph node (*p*=0.048) only, while PPD positivity was significantly related to baseline and clinical features of the participants.

**Conclusion:**

The EBUS-TBNA demonstrated effective utility and safety in the evaluation and diagnosis of intrathoracic TB lymphadenopathy among individuals with compatible symptoms in a country with low-moderate TB-incidence.

**I**n 2020, the World Health Organization (WHO) estimated that tuberculosis (TB) is the most frequent cause of mortality from one infectious organism. Of approximately 10 million individuals worldwide who contracted TB in 2020, only approximately 50% (5.8 million cases) were notified, and approximately 1.3 million individuals who did not have human immunodeficiency virus (HIV) co-infection died from the disease (higher than the 1.2 million deaths reported in 2019); with another 214,000 deaths among persons co-infected with HIV.^1^ Adults accounted for 88% and children for 12% of people with TB. Successful TB treatment requires the combination of 4 anti-TB medications for at least 6 months, but the number of medications may increase in patients with resistance to first-line anti-TB agents. Tuberculosis treatment guidelines in recent times have highlighted the need for a confirmatory diagnosis of infection with *Mycobacterium tuberculosis* as well as their antimicrobial susceptibilities before treatment.^2^

The burden of extrapulmonary TB (EPTB) varies according to region and countries. In European countries, an average of 21.7% of people with TB had EPTB, with the burden ranging from 21.1% to 45.2% among Member States, and some workers reporting a prevalence as high as 48%.^3-4^ In Australia, EPTB represented 34.2% of all notified TB cases, while in Morocco EPTB accounts for 43.5% of all cases of reported TB.^5-6^ A recent analysis carried out in Saudi Arabia estimates that a substantial fraction of notified TB cases are EPTB.^7^ In many of these cases TB involving the intrathoracic and extrathoracic lymph nodes are the most common presentation of EPTB.^3-7^ Obtaining the correct diagnosis in individuals with intrathoracic tuberculous lymphadenopathy (INTBLA) is crucial in order to rule out other differential diagnoses like sarcoidosis, lymphoma and intrathoracic metastasis, and before commencing anti TB drugs. Although mediastinoscopy is the gold standard technique for obtaining tissues samples from the mediastinum, it is limited by the fact that some lymph node stations are not accessible via mediastinoscopy, and the procedure requires surgical expertise and anesthesia.^8-9^ In addition, the procedure is associated with complications like bleeding, damage to contiguous structures, and pneumothorax.

Transbronchial needle aspiration (TBNA), which involves the use of convex probe endobronchial ultrasound that permits real time sampling of the intrathoracic lymph nodes under direct visualization, has now emerged. This diagnostic modality is now a crucial procedure for the sampling of intrathoracic lymphadenopathy. Endobronchial ultrasound-guided transbronchial needle aspiration (EBUS-TBNA) is meritorious for being able to reach most of the intrathorcic lymph nodes under the guidance of ultrasonography – even nodes below 10 mm in diameter, and the procedure-related complications are very small compared to conventional TBNA or mediastinoscopy.^10-11^ Among individuals having lung cancer or sarcoidosis, EBUS-TBNA has demonstrated higher yield and sensitivity when compared to conventional TBNA or mediastinoscopy.^10-11^ Some studies have assessed the importance of EBUS-TBNA during the evaluation of individuals having suspected INTBLA and reported sensitivity rates of 59.3 to 94.0%.^12-15^ There is no data on the usefulness of EBUS-TBNA in the assessment of suspected INTBLA in Saudi Arabia whereas TB burden is relatively low.^1^ Therefore, the aim of this study was to describe the diagnostic utility of EBUS-TBNA in the assessment of suspected cases of INTBLA in a setting with a lower-moderate burden of TB.

## Methods

A retrospective observational study was conducted between 2013 and 2020 at King Abdulaziz University Hospital, Jeddah, Western Region, Saudi Arabia.

All patients with symptoms and signs suggestive of TB (such as cough, weight loss, difficulty in breathing, fever and drenching night sweats) who had intrathoracic lymphadenopathy, had undergone EBUS-TBNA, and had a final confirmation of INTBLA met the inclusion criteria. The exclusion criteria included adult patients with positive acid-fast bacilli in the sputum or bronchial washings, and those with a final diagnosis other than INTBLA like sarcoidosis and bronchogenic carcinoma. The final diagnosis of intrathoracic TBLA was made based on a combination of cytopathological assessment, microbiological evaluation and purified protein derivative (PPD) skin test. All patients participating in the EBUS-TBNA provided written informed consent, and the study approval was granted by the Research and Ethics Committee of the hospital. This report conforms to the Standards for Reporting Diagnostic accuracy studies (STARD) statement and the Helsinki Declaration principles.^16^

All study patients had a prior contrast chest computerized tomography (CT) scan to estimate the size and position of the enlarged lymph nodes before the EBUS-TBNA was performed. The process was carried out under conscious sedation using fentanyl and midazolam. The Olympus (BF UC260FW) EBUS scope was passed through the oral route and allowed for simultaneous endoscopic evaluation and linear ultrasound of mediastinal and hilar structures. The EBUS assessment of the large airways was carried out to identify the lymph nodal station with respect to the major vessels.

During the EBUS-TBNA for each individual, the biggest and most hypoechogenic lymph node was chosen. The special EBUS (21-gauge) needle (NA-201SX-4021) was then passed through the working channel and inserted into the target lymph node guided by the endobronchial ultrasound to perform the TBNA. Once the needle tip was visualized within the lymph nodes, the stylet was pulled out after performing a repeated to and fro motion to remove any bronchial tissues or fibrocartilage.^11-13^ Afterwards, the suction syringe was applied as previously described to obtain samples of the lymph node.^11-13^ Finally, the lymph node aspirates were smeared on glass slides. In most patients, multiple lymph node needle passes were carried out during TBNA sampling.

Once the TBNA was completed, the smears were quickly processed using Shandon Kwik-Diff stain for immediate cytopathologic assessment.^11-13^ The unused samples were stored using appropriate fluid (Sure Path, Germany) for subsequent evaluation in the laboratory. 95% formalin was used to fix tissue fragments and cores, and hematoxylin and eosin (Thermo Scientific, Ohio, USA) was used for staining.

Clinical and radiological properties, as well as pathological findings of caseating granulomas were used to confirm the diagnosis of TB. In addition, aspirates having a positive culture for *Mycobacterium tuberculosis* were considered confirmatory for TB. All subjects with a suspected TB diagnosis also had a PPD skin test, which was considered positive if the induration measurement was ≥5 or ≥10 in HIV-infected or non-HIV patients, respectively. The patients were given 6 months of anti-TB treatment, and they were monitored for 6 more months after completing anti-TB therapy in order to confirm they remained clinically stable and to exclude any false-negative diagnosis of an intrathoracic malignancy.

Pathological features were grouped according to 5 grades as follows: Grade I-necrotizing granuloma, Grade II-non necrotizing granuloma, Grade III-necrosis with non-granulomatous reaction, Grade IV-lymphocytes only, and Grade V-inadequate sample. Provided the background clinical and radiological features, as well as the positive PPD skin test, patients with findings of pathological grades I to III were considered to have a diagnosis of intrathoracic TBLA. Microbiological assessment was considered consistent with a diagnosis of TB if the culture of the aspirates isolated *Mycobacterium tuberculosis*.

The following data was collected from each patient: the patients’ age, gender, nationality, smoking status, HIV status, lymph node stations, lymph node size, number of needle passes during sampling, pathological grades, TBNA culture, PPD skin test, clinical symptoms at presentation, serum levels of C-reactive protein (CRP), erythrocyte sedimentation rate (ESR), red cell distribution width (RDW) and chest CT-scan findings

### Statistical analysis

Statistical analyses was carried out with IBM SPSS Statistics for Windows, version 24.0 (IBM Corp., Armonk, N.Y., USA). Considering the standard definition of sensitivity which is the proportion of the participants with confirmed TB using EBUS-TBNA, and the fact that in the study the prevalence of TB was 100% (meaning all the patients had INTBLA) we were able to estimate the sensitivity of the procedure but were unable to calculate its specificity and predictive values. Categorical variables were reported as proportion, while continuous variables were reported as median (range) or mean±standard deviation. Predictors of a positive culture or a positive PPD skin test were modelled using unadjusted logistic regression analysis. Multivariable regression analysis was not carried out due to inadequate sample size. All tests were 2 sided, and a *p*<0.05 was the cut-off for statistical significance.

## Results

During the time indicated, 31 consecutive presenting individuals who were eventually diagnosed as having INTBLA had EBUS-TBNA at our hospital. Overall, 17 (54.8%) of the patients were over 40 years old, their median age was 44 (range 15-81) years and 25 (80.6%) of them were males. Table 1 summarizes the characteristics of the participants. There were 21 (67.7%) Saudi nationals. The most frequent clinical presentation was coughing in 22 (71%) patients followed by fever in 19 (61.3%) patients and night sweats in 18 (58.1%). Other less common presenting symptoms were weight loss, dyspnea, and hemoptysis as shown in Table 1. Also, 4 (12.9%) of the patients were smokers, 2 (6.5%) had HIV co-infection, and there were 2 (6.5%) patients with abnormal lung parenchyma on chest CT-scan.

**Table 1:** - Characteristics of the study participants.

Variables	n (%)
Total	31 (100)
***Age group (years)***	
≤40	14 (45.2)
>40	17 (54.8)
Median (range)	44 (15, 81)
***Gender***	
Male	25 (80.6)
Female	6 (19.4)
***Nationality***	
Saudi	21 (67.7)
Non-Saudi	10 (32.3)
***Presenting symptoms***	
Cough	22 (71.0)
Dyspnea	9 (29.0)
Weight loss	17 (54.8)
Fever	19 (61.3)
Night sweats	18 (58.1)
Hemoptysis	4 (12.9)
***Smoking status***	
Smoker	4 (12.9)
Non-smoker	27 (87.1)
***HIV status***	
Positive	2 (6.5)
Negative	29 (93.5)
***Abnormal lung parenchyma on CT-scan***	
Yes	2 (6.5)
No	29 (93.5)

HIV: human immunodeficiency virus, CT-scan: computerized tomography scan

**Figure 1:**
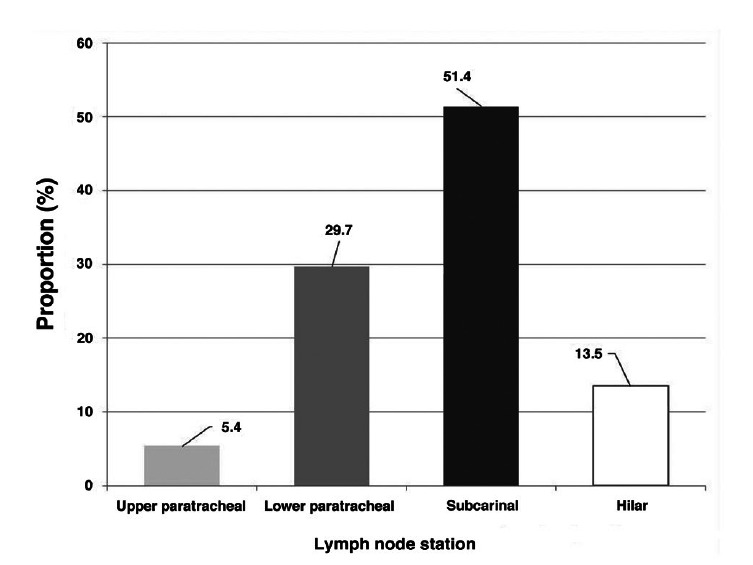
- Lymph node station of endobronchial ultrasound-guided transbronchial needle aspiration.

Based on their clinical presentation and examination findings all the patients underwent EBUS-TBNA. Table 2 summarizes the findings of the EBUS-TBNA of 31 patients with intrathoracic TBLA according to lymph node stations. Samples were collected from 37 lymph nodes. The mean lymph node size was 17.68 ± 5.5mm (median 16.0; range 11-38mm). The most common targeted lymph node in this study was subcarinal (Station No. 7), accounting for 19 (51.4%) of the EBUS-guided sampling, followed by paratracheal (2R and 4R), which accounted for 13 (35.1%), and hilar (Station No. 10) 5 (13.5%) of the lymph nodes (Figure 1).

**Table 2:** - Results for EBUS-TBNA of 37 lymph nodes in 31 individuals having intrathoracic tuberculous lymphadenopathy based on lymph node station.

Lymph node station*	No. of patients with lymph node involvement	Nodes sampled at EBUS-TBNA	Needle passes per station	Nodes from which pathological grades I-III were obtained
2R	5	2	5	2
2L	1	0	0	0
4R	14	11	37	7
4L	3	0	0	0
7	21	19	61	18
10R	5	3	7	2
10L	2	2	5	1
11R	1	0	0	0
11L	1	0	0	0

^*^ According to Mountain-Dresler lymph node map, EBUS-TBNA: endobronchial ultrasound-guided transbronchial needle aspiration

We performed 115 needle passes for 37 lymph nodes. Seven (22.6%) patients had EBUS-guided sampling carried out on 2 or more nodal stations. The overall median number of needle passes per lymph node was 4 (3.8±1.8) (Table 2). The median number of needle passes per lymph node station was 3 (range 1-5; mean 3.2±1.1) among patients who had sampling from only one nodal station, and 6 (range 3-9; 5.7±2.3) among patients who had sampling done from 2 or more nodal stations.

**Figure 2:**
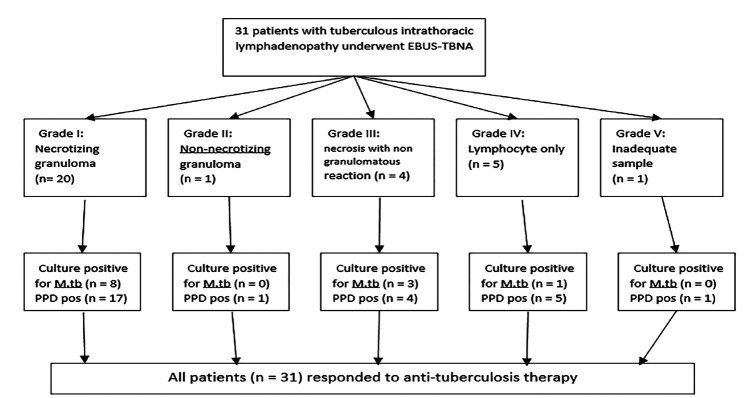
- Flowchart and granuloma grade of subjects with tuberculous intrathoracic lymphadenopathy (TBLA) who underwent endobronchial ultrasound-guided transbronchial needle aspiration (EBUS-TBNA). (*Mycobacterium tuberculosis,* purified protein derivative positive [PPD pos]. Specimens considered inadequate if it contains blood or endobronchial cells with very few lymphocytes.

The EBUS-TBNA confirmed TB disease in 26 patients (83.9%) consisting of patients with a consistent pathological finding or positive culture of Mycobacterium tuberculosis (Figure 2). Pathological analysis had findings consistent with TB in 25 patients (80.6%). Overall, cytopathological analysis indicated that 20 (64.5%) had necrotizing granuloma, 1 (3.2%) had non-necrotizing granuloma and 4 (12.9%) had necrosis with non-granulomatous reaction. Five (16.1%) patients had only lymphocytes from the EBUS-TBNA, and in one (3.2%) patient the sample was inadequate for cytopathological evaluation. Of those 6 patients which EBUS-TBNA showed lymphocytes only or inadequate sample, one (3.2%) had positive culture for *Mycobacterium tuberculosis*, one (3.2%) refused further testing and 4 (12.9%) underwent mediastinoscopy to confirm TB diagnosis. In addition, an assessment of the necrosis grade of the samples revealed that 14 (45.2%) patients had focal necrosis, 10 (32.3%) had extensive necrosis and 7 (22.6%) had none.

**Figure 3:**
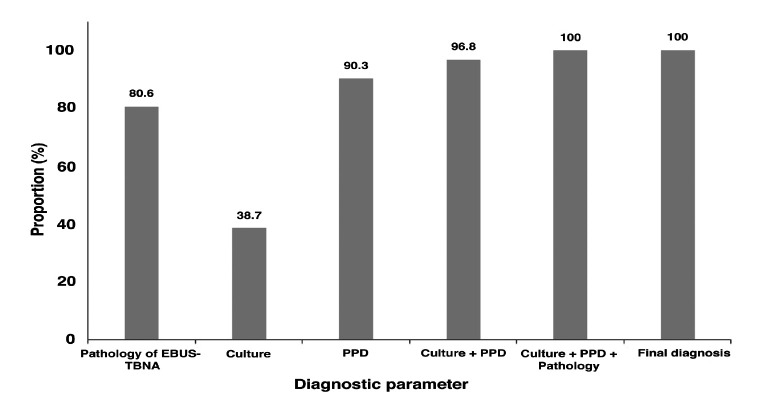
- The accuracy rate of each diagnostic parameter. Culture: culture for *Mycobacterium tuberculosis*, EBUS-TBNA: endobronchial ultrasound-guided transbronchial needle aspiration, PPD: purified protein derivative (tuberculin) test

Culture of the EBUS-TBNA was positive for *Mycobacterium tuberculosis* in 12 (38.7%) patients. Other supportive investigations like PPD skin test was positive in 28 patients (90.3%). Figure 3 shows the diagnostic usefulness and accuracy rate of each modality. Combining culture with PPD skin test, the diagnostic yield reached 96.8% (30/31) while including pathological assessment of the EBUS-TBNA the yield reached 100% (31/31). Overall, the sensitivity of the EBUS-TBNA alone was 83.9%. No major complications, whether procedure-related or anesthetic care-related, were recorded during the EBUS-TBNA procedure.

Table 3 summarizes the factors associated with a positive culture for Mycobacterium tuberculosis of EBUS TBNA samples or PPD positivity in patients who underwent EBUS-TBNA. The EBUS-TBNA aspirate culture positivity was not significantly related to most of the baseline characteristics of the patients or their laboratory parameters (Table 3). However, having a larger size lymph node was significantly associated with culture positivity for Mycobacterium tuberculosis (*p*=0.048). Furthermore, PPD positivity was significantly associated with older age (*p*=0.001), male gender (*p*=0.001), being a Saudi national (*p*=0.004), having a higher CRP (*p*=0.012), higher ESR (*p*=0.003), higher RDW (*p*<0.001), higher pathological grade (*p*=0.003) and higher number of needle passes (*p*=0.002).

**Table 3:** - Univariate analyses of factors that predict positive culture for Mycobacterium tuberculosis or PPD positivity among study participants.

Covariate	Unadjusted OR (95% CI) for culture positivity	*P*-value	Unadjusted OR (95% CI) for PPD positivity	*P*-value
Older age	0.96 (0.92 – 1.01)	0.12	1.04 (1.02 – 1.06)	0.001
Male sex	3.93 (0.40 – 38.70)	0.24	7.33 (2.20 – 24.50)	0.001
Saudi national	1.75 (0.35 – 8.71)	0.49	6.00 (1.77 – 20.37)	0.004
Smoker	1.00 (0.14 – 7.10)	1.00	3.00 (0.31 – 28.84)	0.34
HIV positive	1.00 (0.06 – 16.00)	1.00	1.00 (0.06 -16.00)	1.00
Abnormal CT-scan	0.79 (0.54 – 1.14)	0.20	1.00 (0.06 – 15.99)	1.00
Higher CRP	0.99 (0.98 – 1.01)	0.33	1.06 (1.01 – 1.11)	0.012
Higher ESR	0.99 (0.98 – 1.01)	0.31	1.04 (1.01 – 1.07)	0.003
Higher RDW	0.98 (0.93 – 1.02)	0.32	1.17 (1.07 – 1.27)	<0.001
Pathological grade	0.81 (0.58 – 1.12)	0.20	5.96 (1.85 – 18.23)	0.003
Number of needle passes	0.92 (0.78 – 1.10)	0.37	1.58 (1.19 – 2.09)	0.002
Higher lymph node size	1.21 (1.01 – 1.47)	0.048	1.09 (0.81 – 1.45)	0.57
Sampling of paratracheal nodes	1.13 (0.08 – 15.51)	0.93	-	0.99
Sampling of subcarinal nodes	3.00 (0.26 – 35.33)	0.38	2.33 (0.16 – 34.89)	0.54

OR: odds ratio, 95% CI: 95% confidence interval, PPD: purified protein derivative, HIV: human immunodeficiency virus, CT-scan: computerized tomography scan, ESR: erythrocyte sedimentation rate, CRP: C-reactive protein, RDW: red cell distribution width

## Discussion

This study assesses the effectiveness and safety of diagnosing intrathoracic TBLA by reporting on 31 patients with suspected TB who underwent EBUS-TBNA. Using EBUS-TBNA, we were able to diagnose TB in 83.9% of the cases without any complications or the need for general anesthesia. Further, EBUS-TBNA samples were positive for mycobacterium culture in 38.7% of cases.

The median age of the 31 (80.6%) patients consisting of mainly males was 44 years (range 15-81 years). The sample size, and the sociodemographic characteristics of participants in past studies on EBUS-TBNA, differed according to the indications for the procedure. In the study by Dar et al^14^, 100 participants had EBUS TBNA for the assessment of intrathoracic lymphadenopathy with a mean age of 48.5±16.65 years. In a report by Erer et al,^17^ 28 individuals with mediastinal TB lymphadenopathy had EBUS-TBNA and their mean age was 53.4±15.9 years. In an Indian study,^12^ 100 individuals having mediastinal lymphadenopathy had EBUS-TBNA with a median age of 47.1 years (range 18-83 years).These findings indicate that most patients being investigated for mediastinal lymphadenopathy due to TB or other conditions like sarcoidosis or carcinoma of the lungs are middle-aged, yet the wide age range suggests the need for early diagnosis and treatment.

Subcarinal lymph nodes (Station No. 7), accounting for approximately 51% of the EBUS-guided sampling, and paratracheal (2R and 4R), accounting for 35% of the lymph nodes sampled, constituted the majority of the nodes targeted in this study. These findings corroborate those of other studies assessing individuals with intrathoracic lymphadenopathy. A narrative review among patients with intrathoracic TBLA revealed that the subcarinal and paratracheal lymph nodes were the most frequently sampled nodes during EBUS-TBNA.^17^ Similarly, studies carried out in India, France, Turkey and South Africa also revealed that mediastinal lymph nodes (subcarinal and paratracheal) constituted the largest percentage of the sampled nodes during EBUS-TBNA, while hilar nodes had the smallest proportion.^12,17-19^

The role of EBUS-TBNA has shown sustained growth over the past 2 decades, from its earlier use in lung cancer staging and evaluation of lesions in the mediastinum to a more extensive utility in the diagnosis of intrathoracic lymphadenopathy.^10-11,18^ The EBUS-TBNA sampling carried out among the study patients was adequate in 96.8% of the procedures. Previous studies among patients with intrathoracic lymphadenopathy who had clinical symptoms and signs suggestive of TB or sarcoidosis indicate that sample adequacy from EBUS-TBNA ranged from 89 to 98%.^12,14,20^ This indicates that EBUS-TBNA provides adequate sampling of the intrathoracic lymph nodes to ensure appropriate diagnosis of hilar and mediastinal lesions. In addition, adequacy of samples obtained by EBUS-TBNA prevented the need for mediastinoscopy. An earlier study of individuals with isolated mediastinal lymphadenopathy indicated that EBUS-TBNA averted 87% of mediastinoscopies.^20^ Moreover, in individuals with suspected TB along with compatible clinical or radiological features as in the present study, the use of additional investigative modalities like Ziehl-Neelsen staining for acid-fast bacilli, culture for *Mycobacterium tuberculosis*, PPD skin test, or polymerase chain reaction of the sample for *Mycobacterium tuberculosis*using Xpert MTB/RIF or Cartridge Based Nucleic Acid Amplification test (CB-NAAT) may help extend the utility of EBUS-TBNA during the evaluation of individuals with INTBLA and thereby prevent unnecessary mediastinoscopies.^20-23^

The diagnostic yield involving both histological and microbiological tests based on EBUS-TBNA samples in the present study was 83.9%. This was lower than the overall diagnostic accuracy which was 97.3% among patients suspected with TBLA in South Africa.^24^ *Mycobacterium tuberculosis* was cultured in 38.7% of the EBUS-TBNA samples and 90.2% of the patients had positive PPD skin tests in the present study. Our finding however, varies with those studies assessing the role of EBUS-TBNA during the evaluation of granulomatous pathology (such as TB and sarcoidosis) of the chest. Gahlot et al^12^ diagnosed 71/100 patients who had EBUS-TBNA as having granulomatous lymphadenitis (namely TB in 41 cases and sarcoidosis in 30 cases), with microbiologically verification of TB in 30% of the patients. In another study conducted by Nair et al,^25^ TB constituted the most frequent etiological factor (16/78) in individuals having non-malignant lymphadenopathy; followed by sarcoidosis (11/78). Furthermore, 25% of individuals who were diagnosed with TB had a positive microbiological result.^25^ Put together, this indicates that EBUS-TBNA is an important tool in the evaluation of individuals suspected with INTBLA.

The sensitivity and specificity findings of EBUS-TBNA in the evaluation of suspected patients with INTBLA are variable, but has been found to be very high in some settings.^12-15,24,25^ In the present study, EBUS-TBNA had a sensitivity of 83.9% for the detection of INTBLA. Lucey et al^15^ who explored the utility of EBUS-TBNA among a cohort of individuals with intrathoracic lymphadenopathy of unclear etiology revealed that amongst the subgroup of subjects having TB, EBUS-TBNA had a sensitivity of 59.3% and a specificity of 100%. In a study of 527 individuals with pulmonary conditions and mediastinal lymphadenopathy in Turkey, the sensitivity and specificity of EBUS-TBNA for detecting INTBLA was 87.5% and 98.5%, respectively.^17^ Also, several studies have demonstrated the safety and efficacy of EBUS-TBNA for INTBLA and other mediastinal masses diagnosis.^12-15^ The differences in the diagnostic accuracy reported for EBUS-TBNA during the evaluation of individuals with INTBLA may be due to differences in the burden of TB in the setting where the study was carried out. For example, in Saudi Arabia, there is a lower-moderate burden of TB with TB incidence rate of 20.2/100,000 population in Makkah region, while the setting of the present study (Jeddah) had a rate of 25.4/100,000 population.^26^

The culture positivity rate for *Mycobacterium tuberculosis* of 38.7% observed using the EBUS-TBNA samples in this study is consistent with the culture positivity rates from lymph node sampling reported by similar studies and observed using other diagnostic methods. In a study of 315 suspected TB patients who underwent EBUS-TBNA in the United Kingdom, the culture rate was 35%;^15^ and a study by Erer et al^17^ of 28 individuals who had EBUS-TBNA due to suspected INTBLA in Turkey revealed a culture rate of 46.4%. A study in India revealed a culture rate of 12.5% among individuals with suspected INTBLA who underwent EBUS-TBNA.^25^ The low culture rates for *Mycobacterium tuberculosis* observed may likely be due to variation in the bacillary load of persons with INTBLA; however, the culture rate reported using EBUS-TBNA samples are consistent with the rates gotten with other diagnostic modalities.^10-11^ Further research are needed to evaluate the benefits, if any, of immediate inoculation of the TBNA samples in the TB culture medium. The potential benefits of carrying out rapid molecular techniques, like Xpert MTB/RIF or CB-NAAT system using TBNA samples in order to improve their diagnostic sensitivity deserves further investigation.

The factors that predict positive culture for Mycobacterium tuberculosis of EBUS TBNA samples were evaluated, and only having a bigger lymph node size was significantly related to culture positivity for *Mycobacterium tuberculosis* of EBUS TBNA samples. This suggests that a larger lymph node size may have high bacillary load and may allow better tissue sampling during EBUS-TBNA. A previous study has reported that the presence of necrotic tissue in an EBUS-TBNA rinse fluid increases the diagnostic yield of the samples.^20^ It could be argued that the lymph nodes with necrotic tissue may have higher bacillary load in order to manifest with necrosis and thereby increase the likelihood for the *Mycobacterium tuberculosis* to be cultured.^20^ Furthermore, factors like older age, male grnder, being a Saudi national, having a high ESR, high RDW and high CRP as well as pathological grade and number of needle passes during the EBUS-TBNA significantly predicted PPD positivity in patients undergoing EBUS-TBNA. Some of these factors have been previously documented to be associated with PPD positivity.^27^ In this study, no major complications, whether procedure-related or anesthetic care-related, were recorded. This further emphasizes the safety profile of EBUS-TBNA.

### Study limitations

First, the study utilized a restrospective design, thus we were unable to collect any additional data beyond those documented at the time of the procedure. Second, the sample size was small compared to some previous studies. However, we included all cases of suspected intrathoracic TBLA during the 8-year study period and the sample size could not be increased despite the long study duration. This may be due to the relatively low TB burden in our area. In addition, studies with significantly larger sample sizes compared to this study were multicenter studies involving 4 or more center.^15^ Nevertheless, our findings of the yield and diagnostic accuracy were comparable to other studies. Third, the study was carried out in one tertiary health facility and may not reflect the performance of EBUS-TBNA in a multicenter study involving multiple pulmonologists and cytopathologists.

In conclusion, EBUS-TBNA demonstrated effective utility and safety in the evaluation and the diagnosis of intrathoracic TB lymphadenopathy among individuals with compatible symptoms. The procedure proves its utility across regions with different levels of TB prevalence including relatively low-burden settings. Combined microbiological and cytopathological assessment of the EBUS-TBNA samples need to be carried out, which, combined with other investigative techniques, increases the accuracy of the EBUS-TBNA for the diagnosis of INTBLA. Future research to further address predictors of culture positivity in the EBUS-TBNA samples is recommended.
